# Forearm bisection task suggests an alteration in Body Schema in patients with Motor Conversion Disorders (Functional Movement Disorders)

**DOI:** 10.1192/j.eurpsy.2024.756

**Published:** 2024-08-27

**Authors:** V. Nistico’, N. Ilia, F. Conte, G. Broglia, C. Sanguineti, F. Lombardi, S. Scaravaggi, L. Mangiaterra, R. Tedesco, O. Gambini, A. Priori, A. Maravita, B. Demartini

**Affiliations:** ^1^Health Science Department, University of Milan; ^2^Department of Psychology, University of Milan Bicocca; ^3^Aldo Ravelli Research Center for Neurotechnology and Experimental Brain Therapeutics, University of Milan, Milan; ^4^Unità di Psichiatria SPDC, Ospedale Civile di Legnano, ASST Ovest Milanese, Legnano; ^5^UO Psichiatria 51 e 52, ASST Santi Paolo e Carlo, Presidio San Paolo, Milan, Italy

## Abstract

**Introduction:**

Motor Conversion Disorders (also called Functional Movement Disorders, FMD) are a group of neuropsychiatric conditions characterized by neurological symptoms of altered voluntary motor function that cannot be explained by typical neurological diseases or other medical conditions. In the last decade, several hypotheses have been formulated with respect to their pathophysiology, and a major line of research, trying to integrate psychological, cognitive, and neurobiological factors, focused on the subjective experience that patients feel of their own bodies. However, no study has, so far, directly investigated their Body Schema (the implicit sensorimotor representation of one’s own body) and its plasticity.

**Objectives:**

To investigate the Body Schema in patients with FMD through a paradigm specifically designed to assess their perceived body metrics, through a spatial estimation of body parts length, and to compare their results with the ones obtain on a group of healthy control subjects (HC)

**Methods:**

10 patients with FMD and 11 HC underwent the Forearm Bisection Task, aimed at assessing perceived body metrics, which consists in asking the subject, blindfolded, to repeatedly point at the perceived middle point of their dominant forearm with the index finger of their contralateral hand, and a psychometric assessment for anxiety, depression, alexithymia, and tendency to dissociation.

**Results:**

FMD patients bisected their forearm more proximally (with an increased shift towards their elbow equal to 7.5%) with respect to HC; average bisection point was positively associated with anxiety levels in the whole sample, and with the tendency to dissociation in the FMD group.

**Conclusions:**

FMD patients seem to perceive their forearm as shorter than HC do, which might suggest an alteration of their Body Schema. The Body Schema can go through short- and long-term plastic changes in the life course, mainly related to the use of each body segment; we speculate that, despite FMD being a disorder of functional nature, characterized by variability and fluctuations in symptomatology, the lack of sense of agency over a body part might be interpreted by the nervous system as disuse and hence influence the Body Schema, as deficits of organic aetiology do.

**Disclosure of Interest:**

None Declared

